# An Organic Base without Oxygen

**Published:** 1863-07

**Authors:** 


AN ORGANIC BASE WITHOUT OXYGEN.—M. Rieth has extracted an alkaloid from the bark of the *arariba rubra*, a Brazilian tree. This new alkaloid, which has received the name *aribine*, possesses the remarkable property of containing no oxygen, being the first instance of a solid, non-oxygenous, organic base in nature. The composition of aribine is expressed by the formula C46 H20 N.—*Rep. de Pharm.*

